# Genomics for antimicrobial resistance—progress and future directions

**DOI:** 10.1128/aac.01082-24

**Published:** 2025-04-14

**Authors:** Norelle L. Sherry, Jean Y. H. Lee, Stefano G. Giulieri, Christopher H. Connor, Kristy Horan, Jake A. Lacey, Courtney R. Lane, Glen P. Carter, Torsten Seemann, Adrian Egli, Timothy P. Stinear, Benjamin P. Howden

**Affiliations:** 1 Microbiological Diagnostic Unit Public Health Laboratory, Department of Microbiology and Immunology, University of Melbourne at the Doherty Institute for Infection and Immunity, Melbourne, Victoria, Australia; 2 WHO Collaborating Centre for Antimicrobial Resistance, Doherty Institute for Infection and Immunity, Melbourne, Victoria, Australia; 3 Department of Infectious Diseases and Immunology, Austin Health 3805, Heidelberg, Victoria, Australia; 4 Centre for Pathogen Genomics, University of Melbourne, Melbourne, Victoria, Australia; 5 Department of Microbiology and Immunology, University of Melbourne at the Doherty Institute for Infection and Immunity, Melbourne, Victoria, Australia; 6 Department of Infectious Diseases, Monash Health 2538, Clayton, Victoria, Australia; 7 Victorian Infectious Diseases Service, Doherty Institute for Infection and Immunity, The Royal Melbourne Hospital 90134, , Melbourne, Victoria, Australia; 8 Institute of Medical Microbiology, University of Zurich 27217, Zurich, Switzerland; 9 Microbiology Department, Royal Melbourne Hospital, Melbourne, Victoria, Australia; Houston Methodist Hospital and Weill Cornell Medical College, Houston, Texas, USA

**Keywords:** genomics, antimicrobial resistance

## Abstract

Antimicrobial resistance (AMR) is a critical global public health threat, with bacterial pathogens of primary concern. Pathogen genomics has revolutionized the study of bacterial pathogens and provided deep insights into the mechanisms and dissemination of AMR, with the precision of whole-genome sequencing informing better control strategies. However, generating actionable data from genomic surveillance and diagnostic efforts requires integration at the public health and clinical interface that goes beyond academic efforts to identify resistance mechanisms, undertake *post hoc* analyses of outbreaks, and share data after research publications. In addition to timely genomics data, consideration also needs to be given to epidemiological sampling frames, analysis, and reporting mechanisms that meet International Organization for Standardization (ISO) standards and generation of reports that are interpretable and actionable for public health and clinical “end-users.” Importantly, ensuring all countries have equitable access to data and technology is critical, through timely data sharing following the FAIR principles (findable, accessible, interoperable, and re-usable). In this review, we describe (i) advances in genomic approaches for AMR research and surveillance to understand emergence, evolution, and transmission of AMR and the key requirements to enable this work and (ii) discuss emerging and future applications of genomics at the clinical and public health interface, including barriers to implementation. Harnessing advances in genomics-enhanced AMR research and embedding robust and reproducible workflows within clinical and public health practice promises to maximize the impact of pathogen genomics for AMR globally in the coming decade.

## INTRODUCTION AND HISTORICAL PERSPECTIVES

Antimicrobial resistance (AMR) is a critical and growing global health threat posing a risk to many aspects of modern medical practice. By 2050, it is estimated that approximately 1.9 M people will die every year, and economic losses totaling $1.7 trillion based on disability-adjusted life years lost will be attributable to AMR (1). Understanding the AMR crisis is an incredibly complex issue: it involves diverse molecular mechanisms in multiple pathogens, driven by a range of selective pressures, transmission and amplification in diverse environments from the individual patient to healthcare and community settings, and aspects beyond the human population within the One Health spectrum (2), as well as planetary health concepts with climate change and biodiversity (3).

Pathogen genomics is a powerful technology able to provide deep insights into the mechanisms, emergence, and spread of AMR pathogens. These applications range from the surveillance of transmission between animal, human, and environmental compartments (4), to discovery of new resistance mechanisms (5) to informing clinical and public health decision making based on genomic data (6). Public health surveillance provides the scientific and factual base essential to informed decision making and appropriate public health action (7). The escalation in the use of genomics to study antibiotic resistance has been significant since the early 1990s (8). Advancements in genomics technology, especially massively parallel (next-generation) sequencing, have allowed for rapid and cost-effective sequencing of pathogen genomes, enabling broader application to understanding AMR (9). However, many challenges remain for the broader implementation of genomics for AMR at local, regional, and global scales. Here, we discuss the key requirements for effective genomics-enabled AMR study, progress made in understanding bacterial AMR through the application of genomic technologies, and the barriers and opportunities for implementation at the clinical and public health interface.

### Historical perspectives

The sequencing of the first bacterial genome, *Haemophilus influenzae*, in 1995 marked a pivotal moment in the history of bacterial genomics (8) and paved the way to use genomic technologies to better understand bacterial physiology, metabolism, virulence, and AMR. Subsequent genome sequencing efforts, such as the completion of the *Escherichia coli* genome in 1997 (10), further expanded options for studying antimicrobial resistance mechanisms. Since then, genomic technologies have had a transformative impact on the study of bacterial AMR, accelerated by the advent of next-generation sequencing technologies in the early 2000s.

Sequencing of the first methicillin-resistant *Staphylococcus aureus* (MRSA) genomes in 2001 facilitated research to understand mechanisms of drug-resistance in this major human pathogen (11). This promoted further studies that described the global dissemination of important MRSA clones as well as the early adoption of genomics to understand within hospital evolution and transmission of MRSA (12, 13). Soon after, genomics was used to understand the evolution of MRSA during prolonged clinical infections, providing insights into novel mutational AMR mechanisms (14, 15). Early whole-genome sequencing (WGS) studies also uncovered the role of mobile genetic elements, such as plasmids and transposons, in disseminating resistance genes among bacterial populations (16). The discovery of new AMR determinants was enabled through genomics, such as the discovery of plasmid-mediated colistin resistance in *E. coli* (5) and was further empowered by the application of statistical genomic methods [such as bacterial genome-wide association studies (bGWAS)] (17) to large data sets of bacterial sequences to identify resistance-associated mutations such as *walKR* mutations associated with vancomycin resistance in *S. aureus* (18).

## GENOMIC APPROACHES FOR STUDYING ANTIMICROBIAL RESISTANCE

**Fig 1:**
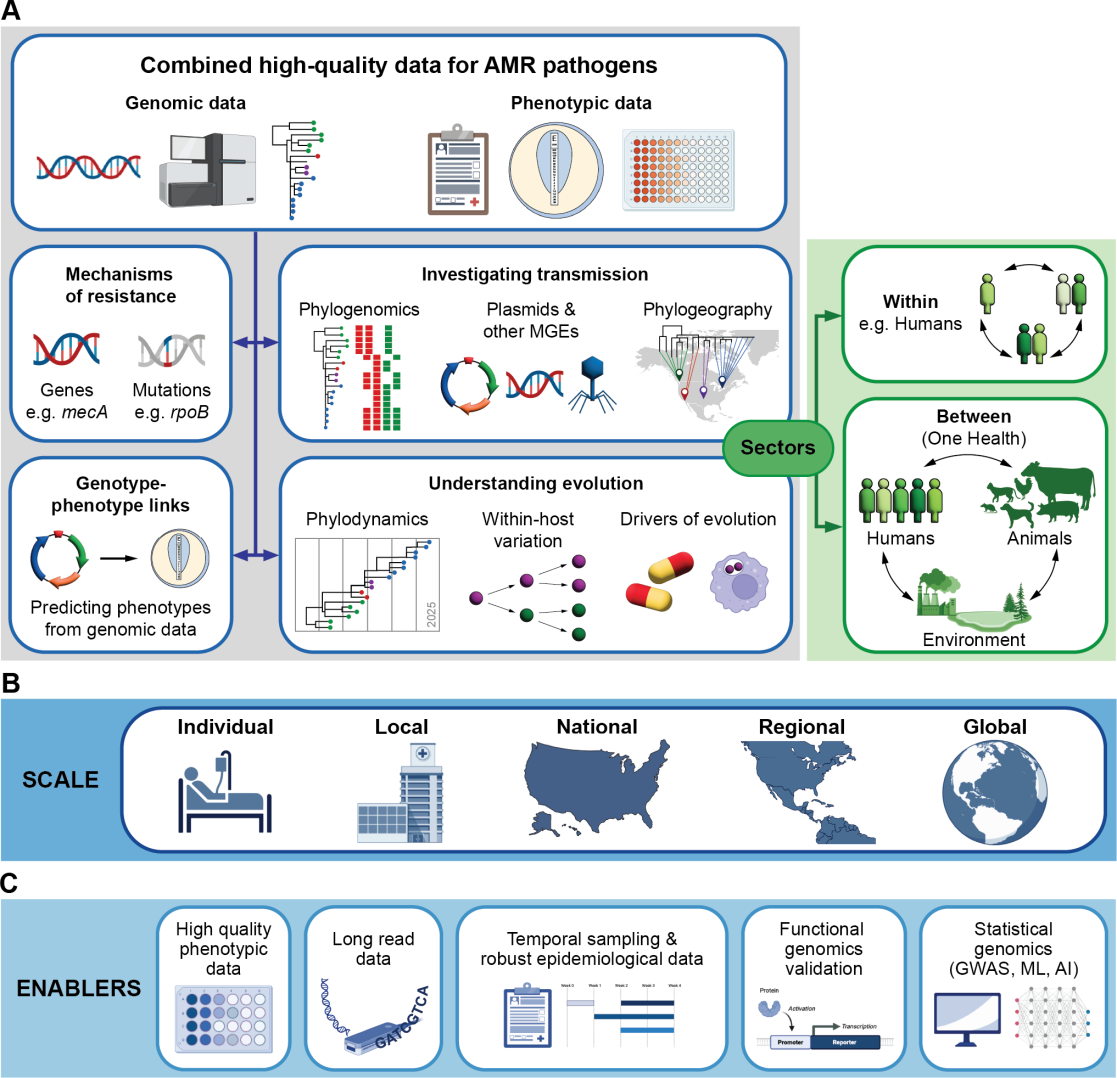
Summary of the inputs and outputs of genomics research for AMR. Highlighted are (A) the relevant data inputs, possible research outputs, and sectors; (B) scale of the investigation; and (C) enablers of high-quality research outputs. MGE, mobile genetic elements; GWAS, genome-wide association studies; ML, machine learning; AI, artificial intelligence.

Genomic approaches can answer many questions relevant to understanding AMR (Fig. 1A), including (i) discovery and detection of the underlying AMR resistance mechanisms (mutations and genes); (ii) determining the link between genetics and resistance phenotypes (genotype to phenotype prediction); (iii) understanding evolution of resistance; (iv) determining the transmission and spread of AMR pathogens or resistance mechanisms with high spatio-temporal resolution; and (v) the connection of AMR within different compartments using the One Health concept. The key inputs into this type of analysis include high-quality, representative, and diverse genomic data, phenotypic AMR data, and relevant contextual epidemiologic metadata.

Investigation and surveillance of AMR using genomics methods have initially focused on independent sectors, such as human health or animal health. The interconnectedness of humans, animals, and the environment is increasingly recognized. Consequently, to overcome these data silos, genomics for surveillance of AMR in a One Health context has emerged. ’One Health’ concept is an important concept to understand transmission and evolution of AMR across different sectors and has been recently reviewed (2). The scale of genomics research and investigation is also relevant and can be applied to detect and understand AMR at an individual [e.g., characterization of an isolate from an individual infection, understanding evolution within a single infection (19)], local (hospital ward or hospital level), national, regional, and global levels (20, 21) (Fig. 1B). The data inputs, analyses, and responses to these analyses will all vary. Table 1 includes selected representative studies demonstrating the different levels at which genomic methods can yield impactful results: large-scale surveillance of AMR pathogens; One Health investigations identifying potential sharing of AMR between humans, animals, the environment, and the food chain; in-depth analyses of AMR transmission within hospitals or community networks; and studies of AMR and its evolution within patients. Recently, even more complex models of AMR transmission beyond the One Health concept have been established, integrating the planetary microbiodiversity and climate-related aspects (3).

**TABLE 1:** Selected examples of WGS being used for AMR investigation/surveillance on different levels (individual patient to international)^*a*^

Use of WGS for AMR surveillance	Location	AMR pathogen	Reference
*International, national, or sub-national surveillance*			
National CRE genomic epidemiology & transmission study	Singapore	CPE	(22)
National surveillance of CRE	China	CPE	(23)
State-wide surveillance of CPE	Queensland, Australia	CPE	(24)
Analysis of carbapenem-resistant *K. pneumoniae* (EuSCAPE)	Europe	CPE	(25)
National surveillance of CPE	Norway	CPE	(26)
National surveillance of CPE	Netherlands	CPE	(27)
Integration of WGS into national CPE surveillance	Philippines	CPE	(28)
National surveillance of 3GC-R BSI	Denmark	3GC-R Gram negatives	(29)
Characterization of 3GC-R *E. coli* from MERINO trial	Australia, New Zealand, and Singapore	3GC-R *E. coli*	(30)
Characterization of ESBL *K. pneumoniae*	Houston, Texas	ESBL *K. pneumoniae*	(31)
Longitudinal surveillance of MRSA in community and healthcare	East England, UK	MRSA	(32)
Genomic epidemiology of snapshot of invasive *S. aureus*	Europe	MRSA	(33)
Early adoption of WGS to characterize *vanA* VREfm	Denmark	VREfm	(34)
*Investigating AMR links between humans and food chain*			
Investigation of links between ESBL-Ec from human and food chain sources	England, Wales & Scotland, UK	ESBL-Ec	(35)
Investigation of CTX-M-14-producing *Salmonella* Kentucky	Europe	ESBL *Salmonella* Kentucky	(36)
Genomic surveillance of VREfm in livestock and humans	UK	VREfm	(37)
Investigating the links between ESBL-Ec and ESBL-Kp in the human environment and food sources	France, Switzerland, Spain, The Netherlands & Germany	ESBL-Ec and ESBL-Kp	(38)
*In-depth analysis of local or inter-facility transmission*			
Resolution of CPE outbreak using long-read sequencing	Maryland, USA	CPE	(39)
Inter-facility transmission of CPE in North Carolina, USA	North Carolina, USA	CPE	(40)
Point-prevalence survey of CPE	Madrid, Spain	CPE	(41)
Analysis of CPE outbreak in neonatal ICU	Nepal	CPE	(42)
Analysis of 3GC-R Gram negatives across three hospitals to inform patient isolation practices	Netherlands	3GC-R Gram negatives	(43)
ESBL *K. pneumoniae* investigation identified polyclonal outbreak in a healthcare setting	Germany	ESBL *K. pneumoniae*	(44)
Prospective study of transmission in high-risk healthcare settings to inform infection control practices	Germany	MDR *E. coli*	(45)
Investigation of MRSA transmission across hospital networks	London, UK	MRSA	(46)
Identification of the source of surgical site infections	Arizona, USA	MRSA	(47)
Surveillance of MRSA in ICU	Cambridge, UK	MRSA	(48)
Surveillance of hospital-onset BSIs suggests community origins	Chicago, USA	MRSA	(49)
Retrospective study of VREfm transmission in a single site	Cambridge, UK	VREfm	(50)
Detailed genomic evaluation of VREfm samples from patients and environment in a single hospital ward	Cambridge, UK	VREfm	(51)
Identification of transposon-mediated outbreak of NDM-1 in a hospital	Germany	CPE	(52)
Study of complex genetic mobility of KPC within a single hospital over years	Virginia, USA	CPE	(53)
*Within patient*			
Emergence of low-level methicillin resistance during therapy	Australia	*S. aureus*	(54)
Emergence of daptomycin resistance during therapy	USA	*E. faecalis*	(55)
Low-level vancomycin resistance	USA and Australia	*S. aureus* (MRSA)	(14, 15)
Large-scale within-host evolution signatures of antibiotic resistance emergence	Worldwide	*S. aureus* *M. tuberculosis*	(56) (57)

^*a*^ CRE, carbapenem-resistant Enterobacterales; CPE, carbapenemase-producing Enterobacterales; 3GC-R, third-generation cephalosporin resistance; BSI, bloodstream infections; ESBL, extended-spectrum beta-lactamase; ESBL-Ec, ESBL *E. coli*; VREfm, vancomycin-resistant *Enterococcus faecium*; EuSCAPE, European survey on carbapenemase-producing Enterobacteriaceae; MDR, multi-drug-resistant; MRSA, methicillin-resistant *Staphylococcus aureus*; ICU, intensive care unit; WGS, whole-genome sequencing.

### Key requirements to enable high quality and impactful genomics-based AMR research and surveillance

As highlighted in Fig. 1C, there are several advances or approaches to enhance the quality and impact of AMR research activities.

#### High-quality genomic data

High-quality genome sequences are an essential pre-requisite for successful AMR research and surveillance. For best analytical outcomes, all genomic data generated should be subject to rigorous quality control (QC) processes. The QC process should include all stages from pre- to post-analytics, including DNA extraction, the sequencing process, and bioinformatic analysis, to avoid misinterpretation and false conclusions (58). When genomic data from public data repositories is used, then this should also undergo QC checks prior to inclusion, as data quality can be highly variable (59, 60). Well-curated databases of high-quality reference genomes [such as NCBI’s RefSeq (61)] are critical to select robust references for comparative genomic analyses (62).

To date, most genome sequences generated have been short-read data (namely from Illumina platforms), yielding accurate but short lengths of DNA (reads) that can then be reconstructed into longer contiguous segments of DNA (contigs). Short-read sequencing is inherently limited in its ability to identify mobile genetic elements such as plasmids, accurately sequence repetitive regions such as insertion sequences, and identify complex structural changes, all of which may contribute to AMR (63, 64). While novel bioinformatic strategies are currently being investigated to improve the utility of short-read data to manage these challenges, long-read sequencing or hybrid assembly of long- and short-read sequences are currently required to accurately resolve these issues (65).

#### Long-read genomic sequence data

Long-read genome sequencing data enhance AMR research and surveillance due to its ability to identify and characterize complex resistance mechanisms. By providing longer reads that span repetitive regions and structural variations, long-read sequencing platforms such as PacBio and Oxford Nanopore offer a more structurally accurate view of the genome. This allows for precise resolution of the genomic context of resistance genes and mobile genetic elements (66). Additionally, long-read sequencing can help in resolving complex genomic rearrangements, e.g., recombination events, inversions and translocations, and copy number variation. Long-read sequencing may also identify novel resistance determinants that may be missed by short-read sequencing methods (67, 68) and more reliable identification of plasmid-mediated resistance and transmission (69). However, while the base calling accuracy of Oxford Nanopore Technology (ONT) sequencing has improved significantly since its introduction, it may still not be sufficient for some purposes, such as deep analysis of genetic variations (70). Additionally, constant changes of protocols and databases make it challenging to operate ONT in an ISO-accredited diagnostic environment with high regulatory demands for stable and well-established technologies. As such, care needs to be taken to maximize read quality (including optimal variant calling) to avoid misinterpretation of long-read sequence data (71, 72). Base calling from methylated DNA can be problematic and can add substantial numbers of incorrect SNP (single-nucleotide polymorphism) calls when the same strains are sequenced by diverse groups, which resulted in disrupted clusters. Whereas PCR preamplification, recent base calling model updates, and an optimized polishing strategy notably improve reproducibility, these issues currently make ONT a non-ideal tool for outbreak investigations across different laboratories (73).

#### High-quality phenotypic data

When investigating relationships between AMR genotypes and phenotypes, it is important to use high-quality phenotypic data, using methods validated according to CLSI or EUCAST standards, to minimize errors or variability (noise) in the underlying data due to the use of non-gold-standard methods. Minimum inhibitory concentration (MIC) data are preferred over interpreted (susceptible/intermediate/resistant) data as the most granular form of phenotypic AST data and can be reinterpreted when breakpoints change. Gold-standard AST methods such as broth microdilution or agar dilution (depending on the organism and antimicrobial) are preferred, followed by other methods producing an MIC, such as gradient strip methods. While other commonly used methods such as disk diffusion produce quantitative data (zones of inhibition) designed to be converted to categorical interpretations (“susceptible” or “resistant”), they are not directly translatable into MICs, potentially limiting their utility in the investigation of genotype-phenotype relationships, or guide discovery of new AMR mechanisms when paired with genomic data. It should be noted that even with the same antibiotic and the same strains, different methods can sometimes yield potentially important differences in MIC determination (74). This needs to be carefully considered when linking genomic data to AMR phenotypes. For most public genomic data sets, there are no integrated AMR data available, and if there are, details on how the AMR profile was generated or what quality control procedures were used are often missing.

#### High-quality epidemiological metadata and appropriate sampling frames

Phenotypic and genomic AMR results are often aggregated as surveillance data or research studies. These data are often used to identify emerging issues requiring infection control or public health response and inform policies such as treatment guidelines. Therefore, available data are strongly biased for AMR or hypervirulent strains, with pan-susceptible strains rarely being sequenced in a structured and systematic effort. To fully investigate and understand AMR on a population (rather than individual) level, data sets must be representative of the target population with little or no collection bias, collected consistently over time and location, and be accompanied by appropriate clinical and epidemiological metadata. Data sets generated from routine diagnostic testing and passive surveillance programs are often subject to biases in healthcare access and may overrepresent severe cases and hospital presentations, resistant pathogens, treatment failures, priority populations, and outbreaks, especially in lower resource and user-pay systems, and may result in estimates which are incorrect or not representative of the target population (75–77). To reduce the impact of these biases, careful targeting of otherwise under-represented cases through active sentinel-surveillance, or routine collection of data from randomly selected patients with clinical syndromes of interest, regardless of clinical need, may be required to supplement data from routine diagnostic testing. Collection and linkage of genomic data to epidemiological and clinical data can potentially enable enhanced understanding of AMR transmission and spread, inform targeted response and prevention activities and risk-factor informed empiric treatment guidelines (6, 78). The data currently available in public repositories are clearly heavily biased, posing significant problems for bioinformatics and machine-learning algorithms (79).

#### Robust detection of resistance mechanisms from genomic data

Detection of AMR determinants using bacterial genome sequences requires (i) a well-curated and diverse database of known AMR determinants and (ii) software tools to identify these determinants. Many software tools exist to address this problem, using several different bioinformatic methods (80). These methods include (i) BLAST matching to database of AMR genes (BLASTN/BLASTP/BLASTX) [e.g., ResFinder (81), ABRicate (82), AMRFinderPlus (83)]; (ii) combined mapping/alignment and targeted local assembly [e.g., ARIBA (84)]; (iii) models identifying homology to existing AMR genes and SNPs in a curated database [e.g., CARD’s Resistance Gene Identifier (RGI) (85), also implemented when no BLAST matches are found in AMRFinderPlus]. Each tool uses one or more AMR reference databases, a catalog of AMR determinants that may include combinations of AMR genes, SNPs, and/or insertions and deletions (indels), which may be restricted to known AMR determinants in certain species or be applied across species. The commonly recognized AMR databases currently available are listed in Table 2. Different tools often generate different outputs with variable interpretation of the presence or absence of specific resistance mechanisms. Using a reference data set but different bioinformatic algorithms to determine AMR genes showed high variability between nine participating labs in an external quality assessment. The authors concluded that comprehensive public resistance sequence databases, full recommendations on sequence data quality, and standardization in the comparisons between genotype and resistance phenotypes will all play a fundamental role in the successful implementation of AST prediction using WGS in clinical microbiology laboratories (86).

**TABLE 2:** Selection of commonly used AMR databases^*a*^

Database	Characteristics
AMRFinderPlus Database (NCBI)	Comprehensive, actively curated database used by NCBI’s AMRFinderPlus software tools Responsible for designation of allele names for new beta-lactamase and tetracycline resistance genes (87)
Comprehensive Antimicrobial Resistance Database (CARD, McMaster University)	Comprehensive database of AMR gene sequences and SNPs Includes another database for antimicrobial resistance ontology; used by CARD’s RGI tool Also actively integrated with NCBI’s AMRFinderPlus database (85)
ResFinder database (Centre for Genomic Epidemiology, Denmark)	Database for the ResFinder tools, including a web-based graphical user interface Actively curated by the Centre for Genomic Epidemiology (Denmark), with an emphasis on prediction of phenotypic resistance (88)
Relational Sequencing TB Data Platform (ReSeqTB database)	Species-specific AMR database for *Mycobacterium tuberculosis*, curated from large global data sets of *Mtb* sequences with phenotypic correlations (89)

^*a*^ NCBI, National Center for Biotechnology Information; SNP, single-nucleotide polymorphism; RGI, Resistance Gene Identifier; *Mtb*, *Mycobacterium tuberculosis.*

While these databases have enabled noteworthy progress in determining resistance mechanisms in genomic data, there are still areas requiring further improvement to meet the needs of different users and use cases. The characteristics of the ideal AMR reference database include (i) comprehensive—include all relevant known AMR determinants; (ii) curated—veracity of AMR determinants (gene sequences or SNPs) is verified, including quality, accuracy, and completeness of sequences; (iii) consistent use of accepted nomenclature; (iv) frequently updated; (v) curators actively respond to queries and new submissions; and (vi) phenotypic correlation available, where possible (90, 91). Several of the larger databases are now very comprehensive, including both AMR genes and mutations, yet there remains underrepresented species, meaning that some species-specific databases are still needed until the AMR mechanism data can be fully curated and incorporated into these major databases. Beyond this, genotype-phenotype correlation and prediction are the next great challenge for AMR databases and tools to tackle, described in the next section.

### Genomic research approaches to advance our understanding of AMR

#### Predicting the phenotype from the genotype

While there have been significant advances in our general understanding of the genetic basis of AMR, establishing direct relationships between phenotypes and resistance mechanisms at the individual strain level has been more challenging, and the “holy grail” of accurately and reliably inferring phenotypes from genomic data has mostly not yet been realized (with some notable exceptions) (92). An important pre-requisite for establishing accurate genotype-phenotype relationships is the need for large, diverse collections of linked high-quality WGS and precise phenotypic data (90). This includes genomic data that has undergone rigorous quality control to exclude events such as plasmid dropout during cultures. For many understudied pathogens and antibiotics, these data are not yet available in the public domain (93). Another important bias is the focus on data sets from high-income data—whereas the greatest burden of AMR likely exists in low- to middle-income countries (LMICs), it is likely that the global genomic diversity is not yet captured by currently available data. Phenotypic data also bring inherent biological and technical variability, and replicative testing across multiple reference laboratories is likely to bring more accurate results. Additionally, changing susceptibility breakpoints and disagreements between EUCAST and CLSI, the two major organizations setting these breakpoints, complicates phenotypic data collections although the use of MIC methods somewhat ameliorates this risk.

Imperfect genotype-phenotype correlations may also reflect complex biological phenomena. While some AMR mechanisms correlate very clearly with phenotypes (such as *mecA/mecC* and beta-lactam resistance in *S. aureus*) (94), most genotype-phenotype relationships are likely more complex and multifactorial. Multiple factors need to be considered for these more complex AMR phenotypes, including variable gene expression, transcription regulation, and interactions between different regulatory genes and AMR mechanisms (95). Ideally, with more high-quality data collections, these predictions can be refined to predict not only binary “Susceptible”/“Resistant” phenotypes, but more nuanced predictions to MIC level, as has already been achieved in some studies (96, 97). The next great challenge for genomic AMR is to bring the data linking genotypes and phenotypes together into comprehensive and consistent databases, with logic for (species-specific) interpretations and their limitations.

Statistical genomics approaches, including machine-learning (ML) whole-genome regression approaches, have shown promise to improve prediction of AMR phenotypes from genomic data through multivariate models (95, 98, 99), although in many cases, they have not been able to sufficiently untangle these relationships to predict phenotypic resistance with a confidence level that is acceptable in clinical and public health microbiology (97). Large-scale bacterial GWAS studies have identified new AMR mechanisms in multiple pathogens (54, 100–104), and novel bGWAS projects including clinical outcome data using general linear models increase the power of these predictions (105, 106). These methods are, however, affected by population stratification, where bias in underlying genetic population structure overcalls links between phenotype and genotype due to lineage effects leading to inflation of false-positive findings for clonal pathogens (107). This bias may be overcome by population structure correction methods to adjust for underlying lineage effects (108, 109). To verify predicted associations, validation in an external data set or using functional genomics (e.g., targeted mutagenesis) is critical. Ultimately, it is likely that a combination of approaches, together with further rigorous laboratory validation, will be best to allow inference of resistance phenotypes from genomic data. Coordination and “crowd sourcing” of large data sets by international organizations may also play an important role in ensuring harmonization of approaches globally (110, 111).

#### Functional genomics validation

The role of putative AMR mechanisms in phenotypic resistance can be validated using methods such as targeted mutagenesis, CRISPR-Cas9 gene editing, and transposon mutagenesis, providing valuable tools for dissecting the genetic basis of AMR (112). Targeted mutagenesis is used to demonstrate the role of point mutations in resistance phenotypes, such as *walKR* mutations in *S. aureus* leading to reduced vancomycin susceptibility (15), or *rpoB* mutations in *Enterococcus faecium* resulting in rifamycin and daptomycin co-resistance (113). Transposon mutagenesis permits random disruption of genes in bacterial genomes and screening for changes in resistance phenotypes, enabling the identification of novel resistance determinants and essential genes involved in resistance mechanisms, as demonstrated in *P. aeruginosa and K. pneumoniae* (114, 115).

#### Insights into the evolution of AMR through genomics

Combining genomic data with targeted wet laboratory methods can further help us understand the evolution of AMR. Research tools such as *in vivo* animal infection and colonization models and adaptive laboratory evolution models offer insights into evolution under selective pressure, new candidate AMR mechanisms (that can then be studied in clinical isolates), and transmission dynamics (54, 116–120).

Genomics of AMR pathogens can provide insights on the evolution of AMR, from the very local scale (single patients), to a global scale. Within-host evolution genomic studies are a powerful approach to identify genetic determinants responsible for the emergence of AMR *in vivo,* including mutations in response to prolonged antibiotic exposure (14, 15, 121, 122) and acquired AMR genes (99, 123). These analyses can be applied to investigate treatment failure in clinical infections, distinguishing between acquired resistance and re-infection with a resistant strain, or through early detection of the emerging AMR mutations (124). On a larger scale, comparative genomics studies of large data sets can also improve our understanding of the emergence of AMR, evolutionary dynamics, and drivers of resistance (125–132).

#### Understanding AMR transmission through genomics

**Fig 2:**
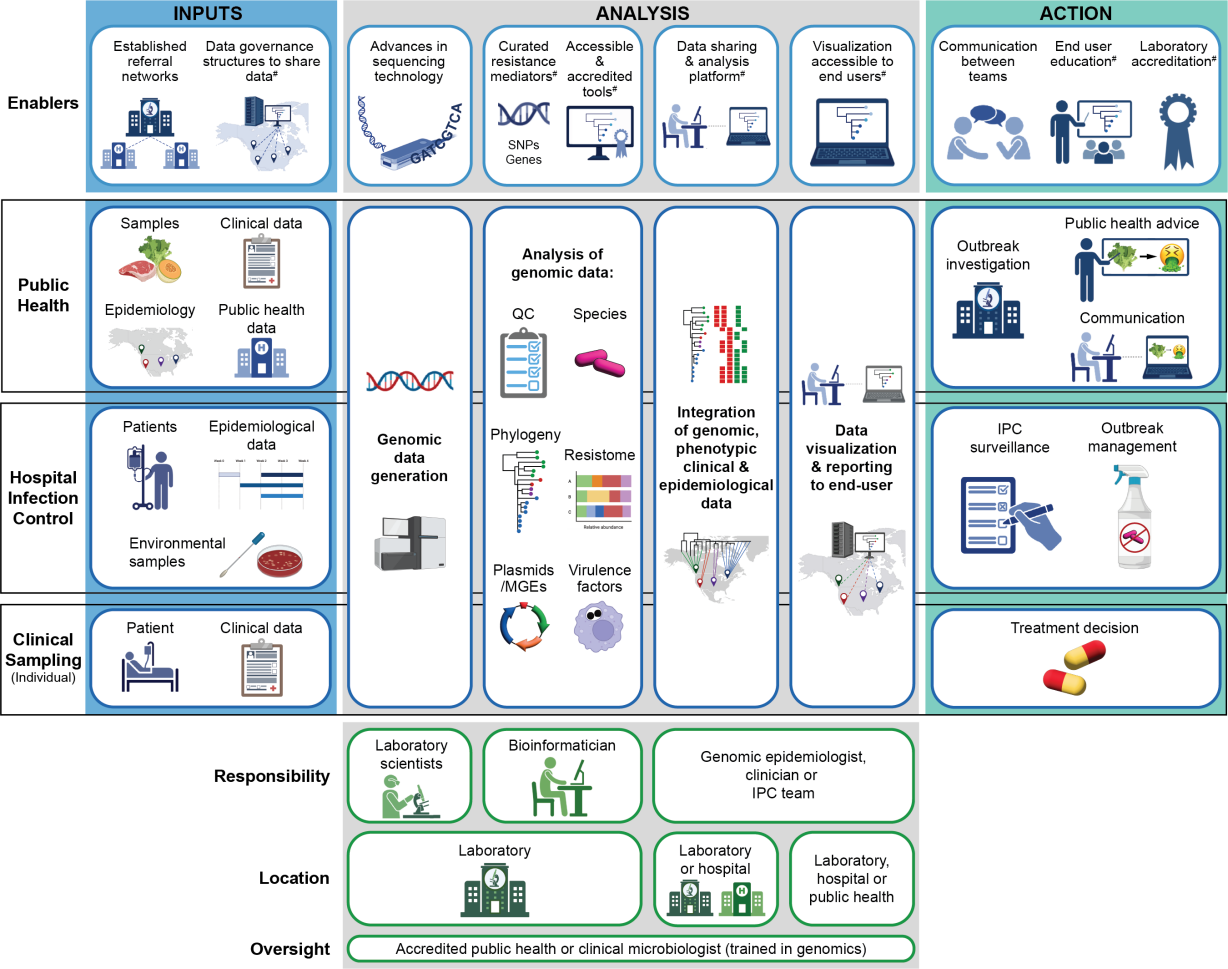
Implementation models for genomics for AMR. The implementation of genomics for AMR into practice can be categorized into different models (public health surveillance, infection prevention and control, clinical diagnostics), each with their own data inputs, analyses that can be undertaken by the relevant responsible team members, analyses that can be undertaken and who is responsible, and the ultimate actions that can flow from these analyses. There are many areas of implementation that require further research/validation or have been identified as key actions requiring further development. These are highlighted by #. QC, quality control; MGEs, mobile genetic elements; IPC, infection control and prevention.

Phylogenetic analysis, involving the reconstruction of evolutionary relationships among bacterial isolates based on genome data, has been critical in tracking the transmission of resistant bacteria and identifying outbreaks of AMR at multiple scales, from healthcare facilities, the community, and nationally or internationally (Table 1; Fig. 2). Phylogenetic analysis can also help identify common sources of resistance transmission and may be useful to assess the impact of infection control measures on containing AMR outbreaks (133). Long-read sequencing has been particularly useful in identifying transmission of AMR due to mobile genetic elements such as plasmids and transposons, which are not usually able to be identified by short-read sequence data (39, 53).

Genomic epidemiology, which integrates genomics with traditional epidemiological data, has further enhanced the surveillance of AMR by providing a detailed understanding of transmission dynamics. Combining genomic data with patient demographics, clinical histories, and epidemiological metadata allows reconstruction of transmission events and provides information that leads to more targeted interventions (Fig. 2). An integrated approach is particularly vital for multi-resistant organisms where understanding transmission can be complicated by long-term colonization (134) and plasmid-mediated outbreaks (135–137). In many published examples, transmission dynamics of an outbreak were only understood after the integration of genomic and detailed hospital epidemiological data, highlighting transmission locations (138), breakdowns in infection prevention and control activities (136, 137, 139, 140), environmental sources of contamination, and the growing risk of international importation and inter-facility spread. This integrated approach has also been implemented within prospective genomic epidemiological surveillance program for carbapenem-resistant organisms, methicillin-resistant *Staphylococcus aureus* (MRSA), vancomycin-resistant enterococci (VRE), and other MROs, to enable rapid transmission detection and response (6).

## APPLYING GENOMICS IN REAL-WORLD SETTINGS: FUTURE DIRECTIONS AND CHALLENGES

In the past decade, genomics has become the cornerstone of AMR surveillance for public health, and the technology is widely implemented in most resource-rich settings. However, the prediction that genomics would revolutionize the practice of clinical microbiology, especially in bacterial pathogen characterization and AMR, has not yet been realized (9). Progress is being made to address barriers to implementation (Table 3), and exciting future opportunities currently exist.

**TABLE 3:** Current benefits of and future challenges for WGS for AMR surveillance

Current benefits
*AMR characterization* Comprehensive view of the entire genome Higher resolution than phenotypic AST or molecular methods Able to retrospectively search for new AMR mechanisms Identification of new therapeutic targets (antimicrobials or vaccines) or diagnostic tests (90, 141–145) *Bacterial typing of AMR pathogens* Much higher resolution than traditional methods Highly reproducible and readily standardized Identification of local or international high-risk clones High-resolution investigation of pathogen transmission dynamics including outbreaks Data portability—comparable between labs, nationally and internationally (145–153)
**Future challenges**
*Laboratory implementation issues* Perceived high costs (154–156) Lack of trained personnel (wet lab, bioinformatics) (157) Newer technology with less well-defined/validated methods (58, 158, 159) Longer turnaround time (TAT) than phenotypic testing or PCR (usually) (142, 160) Bioinformatics bottleneck (80, 161, 162) *Global implementation issues* Timely and representative public genomic data sharing with sufficient metadata Equitable access to genomic sequencing across all countries, generating high-quality, reproducible data Agile platforms and databases to share and visualize data in real-time Appropriate processes to give acknowledgment and intellectual credit to sequencing laboratories (163)

### Rapid diagnostics for clinical microbiology

One of the key potential applications of genomics in clinical practice is “precision medicine” for AMR through the development of rapid genomics-based diagnostic tests that can accurately detect resistance mechanisms in clinical samples—this could incorporate rapid sequencing of bacterial isolates, enrichment broths, or direct metagenomic sequencing from clinical samples and detection of resistance determinants. The choice of platform will ultimately depend on the laboratory and testing requirements; ONT offers the advantage of real-time sequencing and data analysis, while Illumina’s new i100 system provides noticeably shorter turnaround times of less than 8 h from sample to report. In the future, rapid diagnostics based on genomics may guide clinicians in making informed treatment decisions, optimizing antibiotic therapy, and improving patient outcomes by reducing the risk of treatment failure (164).

### Guiding antimicrobial stewardship activities

Genomics may be meaningfully used to guide antimicrobial stewardship efforts by rapidly detecting resistance determinants that may guide therapeutic decisions. For example, rapid metagenomic diagnostic testing methods have demonstrated potential to impact clinical decision making around antimicrobial choices (165), and the increasing use of genomics for inferring phenotypes of *M. tuberculosis* from clinical samples or cultures can identify phenotypic susceptibility or resistance earlier than conventional phenotypic testing, allowing early de-escalation or modification of antibiotic regimens according to results (166).

### “Real-time” sequencing

The early development and use of pathogen genomics took place largely in research settings, with retrospective studies of outbreaks usually suspected by epidemiologic investigations. To reap the benefits of genomics for early detection and control of AMR transmission, “real-time” sequencing implementation is required. Conceptually, this will alert microbiologists, infection prevention and control (IPC) practitioners, or public health agencies of emerging AMR threats or early detection of outbreaks in a timely manner, where interventions are more likely to have a meaningful impact (167). This requires streamlining of sample collection and referral to the laboratory, sequencing and analysis workflows, and reporting and notification processes. Rapid sequencing (often in the hospital’s own laboratory, rather than a centralized laboratory) is an essential pre-requisite to produce clinically meaningful genomic data that can impact directly on patient care but applies equally to infection control and public health genomic surveillance.

Taking this a step further, the concept of prospective WGS, or a “sequence first” approach, is to conduct prospective sequencing of a target AMR pathogen in a specific population, most applicable to healthcare settings. The aim is to identify a small number of transmissions before an outbreak is established, providing the opportunity for early interventions, thus averting potential morbidity, mortality, and unnecessary hospital costs from AMR pathogen transmission (167). A small number of studies have applied prospective genomics for AMR pathogen surveillance in clinical settings. Examples of the potential impacts include evaluation of infection control precautions leading to potential or actual cost savings (133, 139, 154, 168), and identifying genomic links between AMR pathogens in hospitals and the community (32, 169). While feasibility has been demonstrated with a mix of centralized and decentralized sequencing models, several barriers to full implementation currently exist, with costs and turnaround times being of primary concern, with newer studies evaluating more rapid and cost-effective sequencing approaches to optimize implementation efforts (170). Frameworks to define the most cost-effective and targeted surveillance approaches in each setting to avoid unselective, low-impact sequencing efforts will be essential, and education and engagement to promote a multidisciplinary approach with integration of IPC teams will be critical for rapid result feedback and impact.

### Enhancing public health surveillance with AMR genomics

As genomics continues to replace traditional typing methods for public health surveillance, genomic determination of AMR genotypes/phenotypes can be a significant “value add” for the data. Foodborne pathogens such as non-typhoidal *Salmonella* and *Shigella* are examples. Here, the primary role of surveillance is to detect outbreaks and source of enteric diseases; however, genomics has augmented routine surveillance efforts by providing AMR resistance data (when no phenotypic testing was previously performed) or can be used to replace phenotypic testing (171).

In the context of genomic AMR surveillance, genomic databases and repositories are now valuable resources for tracking the emergence and spread of resistant strains across different geographic regions, especially important in the context of international travel and trade (Table 1). Genomic surveillance data can inform public health policies and support international collaborations to address the global challenge of AMR (172). However, more “systematisation and global coordination” is required to optimize AMR surveillance, particularly on the global scale (Table 3). As the WHO promotes and sets the agenda for genomic AMR surveillance through the Global Antimicrobial Resistance Surveillance System (GLASS), there is hope that a globally coordinated approach may emerge (173).

Ultimately, AMR surveillance needs to be agile in ability to identify emerging resistance in real-time across geographical boundaries and able to rapidly characterize new or emerging AMR and identify underlying mechanisms. Several challenges need to be addressed in the near future to achieve this, including public genomic data sharing, equity of access to genomic sequencing across all countries, representative sampling, and agile platforms to share and visualize data in real-time. Initiatives such as the WHO Guiding Principles for Pathogen Genome Data Sharing and the Nagoya Protocol provide frameworks for all countries to work towards (163, 174).

### Issues for genomics for AMR to address in clinical and public health settings

#### Ensuring robust and reproducible results for clinical and public health use

Clinical and public health laboratories are required to adhere to strict quality control criteria for performance, interpretation, and reporting of assays in the laboratory; and in many countries, this means that genomic testing that is being performed and reported needs to adhere to, and be validated to, international scientific organization (ISO) standards (175). This is especially important when the potential impact of the results on clinical and public health actions is high (e.g., changing clinical management of patients, making a public health directive). Given that most AMR databases and software are developed in a research setting (providing highly valuable data and tools), it is important for laboratories to establish the performance of these tools for genomic analyses in the clinical and public health microbiology context (176). Harmonization of methods and interpretations across laboratories, such as external quality assurance programs, is also an essential step to ensure robust and reliable results to inform clinical and public health actions, particularly in the absence of global best practice recommendations and standards (86). The current strict standards for registration of *in vitro* diagnostic devices are also likely to be challenging for genomic AMR testing to meet, requiring laboratories to invest significant efforts to validate these tests for use in patient care.

Even with robust and reproducible processes generating AMR data, communication of results to others who may be less familiar with genomic AMR data interpretation (such as staff in clinical microbiology labs, clinicians, and public health teams) is critical to ensure results are accurately interpreted. Tailored reporting for the audience is essential, incorporating explanatory comments and user feedback where possible, to avoid misinterpretation (171, 177). This should be paired with education efforts to expand the genomic literacy of clinicians and public health teams to maximize the benefits of genomic surveillance and diagnostics (178).

#### Interpretation and clinical reporting

A major challenge in genomics for AMR in clinical and public health microbiology is the interpretation of complex genomic data and the translation of research findings into clinical practice. Translating genomic insights into actionable strategies for patient care, infection control, and antimicrobial stewardship requires an interdisciplinary collaboration between clinical microbiologists, genomics researchers, clinicians, public health officials, and policymakers (179). This requires interventions to optimize implementation at multiple levels, including comprehensive and ongoing education and engagement with health professionals, undergraduates, and trainees; consultation between genomics laboratories and end-users to design appropriate reports to meet the needs of health professionals; developing and optimizing platforms for data reporting and visualization that are fit-for-purpose in each setting; and optimizing genomics workflows and turnaround times to provide timely and actionable data to users (180).

#### Accessible genomics for AMR in low-and-middle-income settings

Antimicrobial resistance disproportionately affects low-and-middle-income countries (LMICs), where limited resources, infrastructure, and access to healthcare contribute to the emergence and spread of resistant bacteria. Resource constraints mean that the appropriate implementation of genomics in this setting for AMR requires careful and context-specific considerations. Inequities in access to genomic technologies and representativeness of genomic AMR data from LMICs clearly exist (181), so understanding use cases in these settings is required. A recent study highlights the utility of genomics for AMR outbreak investigation and response in a resource-limited setting (182) but also clearly demonstrates that “real-time” sequencing and effective communication with IPC and clinical teams is important.

### Future directions and challenges in genomics for AMR

As the world grapples with the threat of AMR, there is no doubt that genomics will continue to have a major and increasing impact on the understanding of, and informing the response to, AMR. One of the future directions lies in the further development of rapid and cost-effective sequencing technologies that can be easily deployed in various healthcare settings, as close to the patient as possible in order to minimize delays and optimize utility for patient care. To ensure sustainability, genomics needs to be targeted to the applications where sequencing is likely to be most cost-effective, hence understanding and quantifying the impacts of genomics for AMR is essential.

The application of artificial intelligence (AI) and ML algorithms in genomics for AMR represents a promising avenue for accelerating the analysis and interpretation of large-scale genomic data sets. AI-driven tools can potentially automate the identification of resistance genes, predict resistance phenotypes, and optimize treatment strategies based on genomic data.

Challenges in genomics for AMR include the need for improved data sharing and collaboration among researchers and public health authorities to facilitate the rapid dissemination of genomic information on resistant pathogens. This will be even more important as the number of laboratories sequencing AMR pathogens is likely to rapidly escalate. Data integration and interoperability across different genomic databases and surveillance systems are essential for tracking the spread of resistance, identifying emerging threats, and implementing coordinated responses. Enhanced data sharing can promote global collaboration, facilitate the exchange of genomic data, and support evidence-based decision-making to address AMR at the regional and global scale.

In conclusion, the future of genomics for AMR holds great promise for advancing our understanding of resistance mechanisms, guiding treatment decisions, and informing public health strategies. By embracing rapid sequencing technologies, leveraging AI, and promoting data sharing and collaboration, the challenges posed by AMR can be more effectively addressed.
